# Participatory action research to pilot a model of mental health service user involvement in an Ethiopian rural primary healthcare setting: study protocol

**DOI:** 10.1186/s40900-019-0175-x

**Published:** 2020-01-08

**Authors:** Sisay Abayneh, Heidi Lempp, Charlotte Hanlon

**Affiliations:** 1 College of Health Sciences, School of Medicine, Department of Psychiatry, WHO Collaborating Centre for Mental Health Research and Capacity-Building, Addis Ababa University, Addis Ababa, Ethiopia; 2 King’s College London, Centre for Rheumatic Diseases, School of Immunology and Microbial Sciences, Faculty of Life Sciences and Medicine, Weston Education Centre, 10, Cutcombe Rd, London, SE5 9RJ UK; 3 King’s College London, Centre for Global Mental Health, Institute of Psychiatry, Psychology and Neuroscience, 16 De Crespigny Park, London, SE5 8AF UK; 4 Centre for Innovative Drug Development and Therapeutic Trials for Africa (CDT-Africa), College of Health Sciences, Addis Ababa University, Addis Ababa, Ethiopia

**Keywords:** Participatory research, service user involvement, action research, Sub-Saharan Africa, Mental health, Patient and public involvement

## Abstract

**Background:**

Involvement of service-users at all levels of the mental health system is a policy imperative in many countries internationally. However, putting policy into practice seems complex; little is known about how best to involve service users and efforts are often criticized for being tokenistic. In low-and-middle income countries, less attention has been given to the roles of service users within mental health systems. The proposed study is part of a larger project intended to develop service-user involvement in mental health system strengthening in Ethiopia. A Theory of Change (ToC) model has already been developed through a participatory approach. This study protocol aims to describe the theoretical background and methods to pilot this model using participatory action research (PAR) and explore participants’ experience of involvement.

**Methods:**

The proposed study will apply a PAR approach situated in critical social theory and conduct a phenomenological case study to find out participants’ experience of involvement. This will be conducted in three stages. The focus of Stage 1 will be to(i) establish a Research Advisory Group (RAG), and Research Participant Group (RPG) at district and primary healthcare facility levels, respectively, and (ii) identify and prioritize potential areas of concern for involvement in the domains of advocacy, service planning and development, monitoring and improving service quality. In Stage 2, we will work with the RPG to develop a plan of action for the selected area. Stage 3 will aim to assist the RPG to implement and evaluate the plan of action. Process indicators and observation will be combined with in-depth interviews with participants to elicit their experiences of involvement. Thematic content analysis will be used.

**Discussion:**

The participatory approach to mental health service user involvement in health system strengthening employed by this study will support the implementation of solutions through locally relevant and contextualized actions. Findings from this study will contribute to the body of knowledge towards understanding the complexity of implementation of service user involvement and refine the ToC model for transferability to similar settings.

## Plain English summary

In order to improve mental health care, it is vital that service planners, managers and health professionals work closely with service users. Service users are experts by experience. They can help to hold services to account and make sure that services reach the people who need them in a fair way. In developing countries, the voices of mental health service users are doubly important to make sure that care is respectful, appropriate and of good quality. But in most developing countries, service user voices are not heard. The aim of this paper is to describe our plans to try out a model of service users and health professionals working together to improve mental health care. The setting will be primary care services in a rural district of Ethiopia. We will set up two groups. Group 1 is called the ‘Research Advisory Group’. The members of this group will be mental health service users, health professionals, officials and community representatives from the district. Group 1 will decide on which problems are most important. Group 2 is called the ‘Research Participant Group’. This group includes service users, their caregivers, health professionals and health managers at a primary care facility. Group 2 will work out how to address the top priority problems. They will then put the plan into action. Together the groups will help to improve mental health care. At the end of the study we will understand more about how services users can be at the heart of improving mental health care in a low-resource African country.

## Background

The importance of involvement of service users and their caregivers (hereafter referred to as ‘service-users’) at all levels of the mental health system has been recognised globally [1, 2]. The concept of involvement (alternatively referred to as participation or engagement) [3–5] is defined as “*a process by which people are enabled to become actively and genuinely involved in defining the issues of concern to them, in making decisions about factors that affect their lives, in formulating and implementing policies, in planning, developing and delivering services and in taking action to achieve change”* [6]. There is explicit international policy direction from the World Health Organization for national mental health systems to empower and involve service-users in mental health advocacy, policy, planning, legislation, service provision, monitoring, research and evaluation [7, 8]. The same directive has become a policy imperative and is therefore firmly embedded in policy documents of many high income countries [1, 9].

In low-and-middle income countries (LMICs), where more than 80% of service-users are living [10], there is less prioritization and government support for either mental health care provision or involvement of service users [10, 11]. In many of these countries, there are no policies and laws to direct mental health programs and/or the policies and laws are not aligned with human rights recommendations (e.g., social care, participation) or are poorly implemented [10, 11]**.** Service-users are exposed to stigma and discrimination [12, 13] and have several unmet needs [13], exemplified by suffering of illness and disability [14], impoverishment [15], premature mortality [16, 17], and human rights abuses(e.g., being chained or kept in isolation) [12, 18]. Studies suggest that service-user involvement can also protect and promote human rights [19, 20]. In LMICs, service-user involvement has been widely recommended as an essential ingredient to strengthen weak mental health systems [21, 22], increasing the likelihood of scale-up of appropriate, quality mental healthcare [23, 24] and thereby reducing the treatment gap [8, 25]. However, little is known about how best to optimize and lasting involvement of service-users [5, 26]. Service-users are often excluded (rendered invisible and voiceless) from their rights to meaningful participation in decisions that have direct impact on their lives [18, 26, 27]; and are at risk of being left behind during efforts to expand universal health coverage [28].

Methods for service-user involvement have been criticized for the lack of a participatory approach/inclusivity, and being unable to move beyond a tokenistic mode of participation [1, 2, 26, 29]. One promising approach to address these criticisms is Participatory Action Research (PAR). The PAR approach is highly conducive to enable marginalized people (in this case people with lived experience of mental illness) to be meaningfully involved in areas of concern to them through developing their capacities and address more holistically the complex factors that hinder their involvement [30–32]. Our recent systematic review (Abayneh et al., in progress) found that PAR is a well-established approach to involve service-users within mental health systems in high-income countries; however, there are few similar studies from LMICs.

### Objectives

The proposed study is informed by a larger project, ongoing since 2014, intended to develop service-user involvement in mental health systems strengthening in Ethiopia. The aim of this paper is to describe the theoretical foundations and methods for a PAR case study of piloting a Theory of Change model for service-user involvement in mental healthcare in a primary health care setting in rural Ethiopia. The specific objectives are to:

- Identify, prioritise and select an area of concern with respect to the integration of mental health into primary healthcare as a focus for involvement of service-users, from the perspectives of service-users, caregivers, health professionals/managers and other key community stakeholders.
- Develop plans for action for the selected area of concern.
- Assist service-users, caregivers, health professionals and managers in the implementation of the plan of action.
- Evaluate the process of, and explore the experience of, involvement of service-users, caregivers, health professionals and managers in the PAR activity.

### Theoretical Foundation

Historically, mental health service-users have been excluded from participation in many mainstream social structures, disqualified as knowers and in knowledge production, because they are construed as irrational, unreasonable, incoherent, lacking in insight, deviant from standards of normalcy, unpredictable, unsafe to themselves and others, victims or deficient of mental capacity [33–35], and considered to have a flawed or spoiled identity [33, 36]. Within the mental health system, knowledge gained through formal education is often more highly valued than the experiential knowledge of service-users gained through lived experiences [33, 37, 38]. Although health professionals and researchers have important perspectives on science and practice, service-users can contribute their unique expertise as individuals with lived experiences of their condition and as a recipient of the healthcare services.

When service-users do have contact with mental health services, the system can hinder [39] or deprive them of any real chance to participate, or their input may be devalued in decision-making with respect to aspects affecting their lives [29, 33, 40]. Health systems value and legitimize service providers to act in the “best interest” of service-users [36, 41]. Service-users may have little control over either the nature of the services they receive or the evidence base that legitimizes these services [33, 37, 42]. They have described the exclusion and neglect of lived experiences from knowledge production as ‘false and potentially dangerous views of the world’ [43] and have highlighted the crucial contribution that their ‘experiential knowledge’ has to bring to the ‘evidence table’ [38, 44, 45], including constructing alternative narratives of experiences and new forms of knowledge [46, 47]. There are also a range of international studies supporting the desirability of lived experience and knowledge for health systems strengthening [48, 49]. However, pervasive stigmatizing attitudes and discrimination [12, 50, 51] at multilevel tend to disqualify service-users from full social acceptance, marginalize them and hinder their active involvement [33, 36, 52].Furthermore, because of these negative attitudes and practices, service users may experience powerlessness, consider themselves as ‘lesser citizens’ or feel unable to act, worthless and incompetent [53, 54], commonly described as “internalized oppression” [55].

Given these factors, the authors argue that service-user involvement needs to be approached within a critical paradigm [56, 57]. More specifically, we choose to ground the proposed study in critical social theory (CST) [58] with focus on Habermas’s theory of communicative space and action [59].The choice of CST has significance in several ways for the proposed study. First, CST can offer a ‘communicative space’ required to create fora for service-users and other stakeholders to engage in dialogue to reach inter-subjective agreement, mutual understanding, and consensus to guide deliberate, and collaborative social action [60–62]. Communicative space, as employed in this protocol, refers to the spaces in time and place where service-users, caregivers, health professionals/managers and other key stakeholders come together in the PAR process [62, 63], and within created social arenas (e.g., mutual recognition, trusting relationship, reciprocal perspective taking, a shared willingness to consider one’s own conditions, learning from each other, reaching common ground for action, sense of agency) [64, 65].

Second, CST guides towards recognizing the social, economic, political, and historical contexts that shape human thought and action, and the social structures that have historically served to oppress certain groups in society(e.g., persons with lived experience of mental health conditions) [66–68]. CST can give clues about how to transform social relations of power and enable service-users through (a) expose injustice (through critical analysis and questioning of long-standing established rules, beliefs and practices and conceptualizations about service-users); (b) challenging relationships of domination that exist within the lives of service-users, and allowing them to engage on an equal footing by bringing service-users, health professionals and health administrators to collaborate on a common issue [60–62], and (c) creating opportunities for service users to gain experiences of emancipatory knowledge and greater awareness about their situation, break attitudes of silence, gain confidence and abilities, open themselves up to new ways of understanding, take effective action to alter unjust conditions and structures [69], to formulate alternative stories that are empowering [65, 70], and gain more control over their situation [68, 71, 72]. Third, CST is based on a capability/strength-based approach of participation [69],engages directly with service--users [73] and acknowledges service-user knowledge as valid, encouraging mutual recognition and sharing of perspectives [59, 73].

## Methods

### Setting

The proposed study is part of a larger project that was instigated as part of the ‘Emerging mental health systems in low- and middle-income countries’(Emerald) project, which investigated the health system requirements for successful implementation of integrated mental health care in six LMICs(Ethiopia, India, Nepal, Nigeria, South Africa and Uganda) [74, 75]. The study will take place in Sodo district, a rural district located in the Gurage Zone of the Southern Nations, Nationalities and Peoples’ region, about 100 km south of Addis Ababa. The district had a population of 161,952 people in 2007 [76]. Around 90% of the district population reside in rural areas and are reliant on subsistence farming [77]. The district population is predominantly composed of the Gurage ethnic group and followers of Orthodox Christianity. The official language of the district is Amharic [76]. There are 58 sub-districts or *kebeles* (the smallest administrative units with 2000 to 5000 people each), which are both geographically and climatically diverse. There is one primary hospital with an outpatient psychiatric service (run by a psychiatric nurse) in the main town and eight health centres, four of which are located within the three towns of the district. The primary hospital and all eight health centres have functioning mental health services using a task-shared model of care. Each health centre serves about five sub-districts, comprising a population of about 25,000–40,000 people [78]. Each sub-district has a health post (lowest statutory healthcare facility)*.* The health posts are staffed by a pair of community health workers called health extension workers (HEWs). The HEWs are high school graduates with one year of training in sixteen packages of care which cover four main areas: disease prevention and control, family health, hygiene and environmental sanitation, and health education and communication [79].A minority of HEWs have received training in mental health as part of their upgrading to level IV.

Sodo district is the research and implementation site for the PRogramme for Improving Mental health carE (PRIME) [80, 81]. As part of PRIME, primary care staff in Sodo district have been trained to deliver packages of care for people with mental health conditions, including prescription of antipsychotic medication, follow-up, limited adherence support, basic psycho-education and community awareness-raising of mental illness [82]. PRIME established a multi-sectoral community health advisory board with representatives from key members of the district leadership (security, gender office, women and youth affairs, religious affairs and education), the community and service users and caregivers, and was chaired by the head of the district health office [82]. The CAB met twice a year to oversee and advise PRIME [83].

### Design

This section describes the research design, rationale and stages of the proposed study. We propose to use a PAR approach [84] and a phenomenological case study [85] to explore participants’ experiences of involvement. In this study, drawing on work by Rouleau et al. [86], and Nelson et al. [87], PAR is defined as the: (i) valuation, mobilization and legitimization of service user experiential knowledge of living with a particular health condition(e.g., mental illness); (ii) conduct of research that focuses on service users’ concerns, participation, and outcomes; and (iii) active partnership among a variety of stakeholders/actors(e.g., researchers, health professionals, decision makers, organizations, service-users) [86], for the purpose of taking action and making change [87]. PAR is the approach of choice for the proposed study for several reasons.

First, PAR has a collection of research methods (epistemological pluralism) [88] that is uniquely suitable to address complex problems (such as service-user involvement [3, 89]), build evidence in areas that lack an empirical evidence base and find practical solutions in the areas of health systems strengthening, implementation research, various health and social care settings [88, 90, 91]. PAR has been increasingly said to be more robust than other approaches because the process (a) simultaneously generates knowledge and initiates actions informed by that knowledge [73, 92, 93], (b) makes knowledge accessible and relevant to stakeholders to underpin change [93, 94], (c) relies on a commitment to bring together theoretical and methodological expertise and the practical knowledge of non-academic participants (‘creates self-critical communities’ [95, 96]), (d) shares leadership and resources to address issues in specific systems [67, 97], and (e) enables co-design of culturally appropriate and effective interventions, their implementation and collaborative evaluation of impact [60, 95].

Second, although many programme theories articulate intended changes [98], engage with the complexity of interventions and provide a framework to guide action, monitoring and evaluation [99], there is little empirical evidence of how theories can be applied in practice [100–102]. Hence, there have been calls for PAR in theory-based implementation and evaluations [103–105]. Early integration of Theory of Change (ToC) and PAR during planning and implementation is recommended [103, 106, 107]. To the best of our knowledge, no one has yet offered a description of how to combine ToC and PAR to apply service-user involvement in mental health systems. We attempted to address this gap by taking cues from the evidence base on combining ToC and PAR from other disciplines (e.g., agriculture [100, 108], education [102], development studies [109], programme evaluation [110, 111] and implementation sciences [112]. For our proposed study, combining ToC along with PAR is a promising approach for several reasons:

A) At the core of both ToC and PAR is a concern with how and why change takes place. Both anticipate a range of positive changes/outcomes [96, 100] including: individual level outcomes(e.g., advancing participants’ personal and collective sense of agency, social networks) and community level outcomes(awareness raising, stigma reduction, and strengthening community capacity, collaboration) [54, 56, 113]. ToC provides a strong heuristic device for deeper understanding of the implementation context [100, 112, 114], guides the direction of change and how to achieve the intended transformation, defines collaborative outcomes and surfaces the various layers of interventions with underlying assumptions, and ensures that different perspectives of participants are reflected in the design [111, 115, 116]. However, that alone may not be sufficient to support the actions required to achieve implementation [102, 107, 108]. This gap can be balanced when ToC is combined with PAR, because the cyclical nature of PAR (iterative cycles of reflection, planning and acting), facilitates learning about what, how and why change is unfolding [103, 108, 111].
B) In practice, ToC and other programme theories may have a problem of reach, i.e., they do not explicitly consider issues of inclusivity and there is little acknowledgement of the way in which power operates to affect the building of collaborative capacity or how this notion needs to be addressed to enable stakeholder participation [106, 107, 117].Without such explicit consideration of power dynamics, ToC approaches may inadvertently reinforce a hierarchical relationship between stakeholders and privilege the perspectives of those in power (e.g., policy makers, professionals) and downplay or even disregard entirely the views of others(e.g., service-users) [116, 118, 119].

These drawbacks may be minimized through the emancipatory and critical theoretical foundation of PAR that seeks to explicitly and intentionally work with a range of stakeholders, including those historically oppressed, disempowered, vulnerable and marginalized groups (e.g., service-users) [62, 107, 112]. The participatory commitment of PAR provides space for diverse forms of expertise and promotes understanding of the different life-worlds of participants [32, 87, 120]. Working in a collaborative and non-hierarchical manner may facilitate deeper understanding of how implementation can be achieved [56, 84, 100, 112, 121]. Furthermore, the dialogue and critical reflection incorporated within PAR enables participants to challenge the status quo of professional-dominated health systems, dismantle unequal power relations between service users/caregivers and those within health systems and society and create fruitful communication [121–123].

C) PAR also rejects objectivist assumptions that distance the researcher and the participants from one another [121, 123]. The close proximity of working can promote inclusion and confront engrained stigma and prejudices [32, 120, 124]. In line with the contact hypothesis [125] and social contact theory [125, 126], positive contact between service-users, health professionals and others within the health system, e.g. by giving them equal status in pursuing common goals, can foster mutual understanding and reduce stigmatizing attitudes [127, 128].

### Sessions and stages of the proposed study

**Fig. 1:**
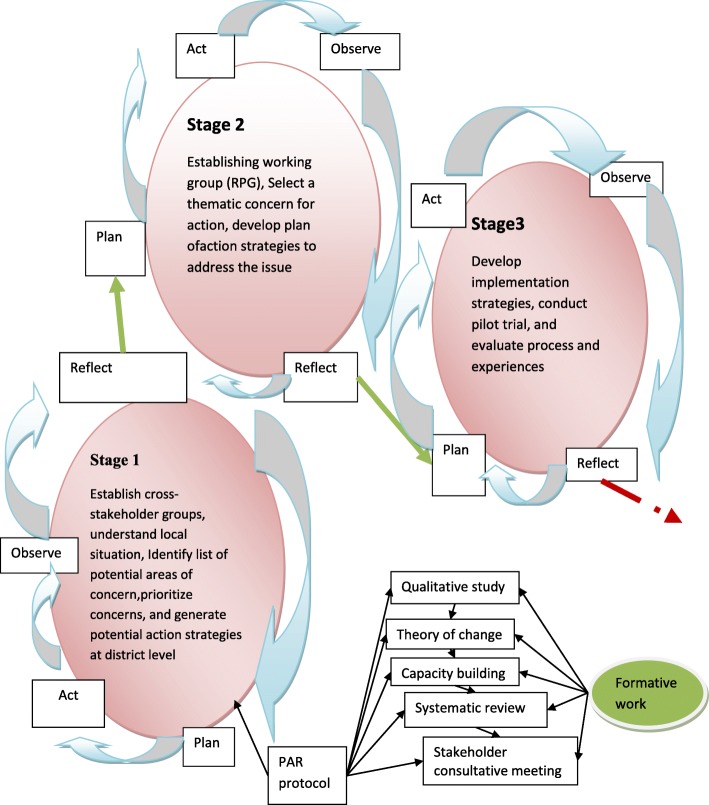
Formative works and stages of the Proposed Study

The study procedure will take place in three stages, with cyclical recurring activities involving planning, acting, observing and reflecting, informed by the model proposed by Kemmis and McTaggart [129] (See Table 2 for proposed sessions plans, stages and activities). The three stages are: i) Establishing of groups, identification and prioritization of thematic concerns, ii) Planning of action, and iii) implementation (See Fig. 1, for summary of stages). We anticipate that the participants will need to meet for at least seven weekly sessions for two to three hours. The first two sessions will take place at district level at Buie town, the capital of Sodo district, and the other five sessions within the primary health care facility and will be conducted in Amharic language.

## STAGE 1: establishment of groups, discussion of foundational studies, identification and prioritization of concerns

### Formation of cross-stakeholder groups (planning)

For the proposed study, we aim to maximize participation of diverse representatives of the local community through establishing two multi-stakeholder groups that will collaborate and be involved within the research process: (i) a Research Advisory Group (RAG), and (ii) a Research Participant Group (RPG).

#### Research Advisory Group

The importance of involving a Research Advisory Group (RAG) was recognized early during the larger study. Although our plan was to establish a new RAG, after our discussion with Sodo district health officials, we agreed to work with the existing community advisory board(CAB)that had been established to oversee efforts to expand access to mental health care in the district (working with PRIME) [82]. As part of the larger study, the Sodo district CAB has participated in the co-production of the ToC for mental health service-user involvement and contributed to a community stakeholder consultative meeting. To ensure a feasible working group size, for the RAG we will purposively select20 participants from the larger CAB. Decisions on who to select will be made collectively with district officials involved in the mental health care programme, based on pre-specified criteria (See Additional file 1for inclusion criteria). In addition we will ensure gender representativeness of participants.

The RAG will play several roles in the proposed study, including: (i) oversee and advise on priority problems for improving mental health care from their local community perspectives, (ii) provide a conduit between the Research Participant Group(RPG) and the community to ensure that the research findings are put into action and disseminated in their local context; (iii) create a strategy to enable an empowering environment(e.g., through resource mobilization) for service user involvement,(iv) facilitate further consultation and community involvement for service user mobilization and empowerment, and (v) advocate for the protections of rights of service users. The RAG will meet three times during the course of the research in Sodo district: two half-day meetings during Stage 1 and once in Stage 3.

#### Research Participant Group

A Research Participant Group (RPG), comprising of up to 12 participants (mental health service-users-*n* = 4, caregivers-*n* = 4, and health professionals and health facility managers-*n* = 4), will be convened at a health facility in Sodo district (See Additional file 1for inclusion criteria to guide purposive selection of participants). As our research objective is not specific to certain mental health conditions, and to increase the social validation of the study objectives, procedures and outcomes [130], we will included service users with psychosis, depression, epilepsy, and alcohol use disorder. We also try to balance gender representation of participants. The RPG will participate throughout the research process in (i) identification and prioritization of priority problem areas, (ii) identification of specific areas of concern at the health facility level; development, implementation and evaluation of an action plan, and (iii) validation of the research process and local dissemination of the findings of the study. The principal investigator and a research assistant will act as facilitators of the process of prioritization, design, conduct and dissemination of activities of the research study with a view to empowering the RPG at each stage in the PAR cycles.

### Discussion of foundational studies, identification and prioritization of potential areas of concern(action)

This session will include two sets of activities including [1] presentations and discussion about foundational studies, and [2] identification and prioritization of potential areas of concerns as briefly detailed below.

#### Presentation and discussion about formative works

The development of this protocol was informed by formative work: (i) a qualitative study, (ii) development of a Theory of Change model for service-user involvement, (iii) capacity building training, (iv) a systematic review, and (v) a community stakeholder consultative meeting (See Fig. 1) in a larger project intended to develop service-user involvement in mental health system strengthening in Ethiopia. In Stage 1 of the proposed study, based on the findings of the formative works, a half-day consultative workshop with representatives from stakeholder groups will be conducted to ascertain the situation of service user involvement in mental health system strengthening in the study site in relation to the global situation. The intention of this protocol is not to report the details of the foundational studies; rather we present a brief overview of the ToC to inform readers about how that informed the development of this protocol.

#### Theory of Change

As part of the larger study, a generic ToC for service-user and caregiver involvement in mental health system strengthening in Ethiopia was co-produced with stakeholder groups, including service users, caregivers, psychiatrists, researchers, and statutory and non-statutory community representatives. The ToC helped to make explicit the hypothesized pathways to achieve the long-term outcome (derived by consensus) of “improved physical and mental health, economic productivity and social inclusion for service users, and improved life satisfaction, including economic capacity for caregivers”. The ToC also allowed identification of necessary preconditions for success, programme levels for an intervention (service user/caregivers, health facility and community), indicators of success, assumptions underpinning the pathway and the types of interventions needed. In the ToC, capacity building training for service users, caregivers, and health professionals/managers, PAR with stakeholder groups, inter-sectoral collaboration, and service-user mobilization were identified as programme interventions to enable service user involvement to achieve the long-term outcome.

The co-production of the ToC with diverse stakeholder groups and the embedded PAR in both the design and implementation of the interventions will enable the ToC to be responsive to local needs [131–133]. However, in the same way that most ToCs are comprehensive road maps for the implementation of a programme [102, 103, 107], our one was also generic and cannot show the specific target of action for service user and caregiver involvement. Service users can potentially be involved in each domain of the mental health system (service planning, service development and delivery, service quality improvement, education/training, service promotion and advocacy); however, there is no evidence-based algorithm to determine how to prioritize the domains. The embedded PAR as an intervention component in our ToC can help to identify and prioritize problems, the specific targets of action as well as the domains of mental health systems, and develop a plan of action based on unique local contexts and strengths by involving service users and other key stakeholders [102, 107, 134]. Therefore, to specify the ToC interventions fora primary healthcare setting, we will conduct a half-day participatory interactive workshop involving stakeholder groups (See prioritization section).

#### Identification and Prioritization of Thematic Concerns (Action, Observation and Reflection)

In the proposed study, we will use PAR to initiate a one-day priority setting exercise by bringing together service users, caregivers, health professionals/managers, and CAB members (including RPG and RAG) to generate a list of their top priority problems for research in involving service users in mental health system strengthening in Sodo district. The study will be informed by guidance from the James Lind Alliance (JLA) [135] to ensure a balanced, inclusive and transparent process for priority problems identification, and the Nominal Group Technique (NGT) to establish consensus, prioritize and rank the thematic concerns [136–138]. The JLA approach enables us to create an environment that encourages open discussion, respect for diversity and clarity of thought, and also has been used to identify research priorities in several areas including mental health [135, 139].

For the proposed study, potential priority problems for involvement of service users in mental health systems improvement will be identified and short listed by stakeholder groups in a 1-day workshop in Sodo district using the five step JLA process [135](see Table 1 for details of the steps). Given the complex nature of service user involvement within the mental health system [3, 5, 89], and low levels of experience of service users, caregivers and other community stakeholders working together within mental health systems in Ethiopia [140], the principal investigator will provide a list of potential priority problems (questions) extracted from the foundational studies and evidence review (See Additional file 2) to prompt discussion and enable participants to choose problems of relevance to their local context. To reduce the possibility of bias and influence by the potential priority problems/questions, the principal investigator and facilitators will encourage participants to reflect upon the sample priority areas, to modify or drop the potential thematic concerns provided and add their own thematic areas that are most important for themselves.

**Table 1:** Procedures to thematic concern identification and prioritization

Stages	Description
Step 1. Establishing the priority setting Partnership and defining scope	A cross-stakeholder groups(*n* = 20–25)including RPG and RAG will be selected with maximum variation comprising of relevant statutory and non-statutory organization representatives and individuals that can reach and advocate for, mobilizing resource, empower and support service users for involvement in mental health systems strengthening. The stakeholder groups will be invited to a half-day consultative meeting and discuss on the findings of foundational studies in Phases 1&2 about service user involvement so as to raise awareness, create the need for collaboration, and define the scope of the study for future action.
Step 2. Gathering and identifying questions	The cross-stakeholder groups will be invited in a 1-day thematic concern identification and prioritization exercise at Sodo district. The participants will be divided into four homogeneous groups (service user, caregivers, and health professionals, community stakeholders) so that the participants are comfortable voicing their opinions. Each group separately will be asked to list as many priority questions from their own perspectives perceived as the most important challenges to be addressed for service user and caregiver involvement in mental health system improvement. Facilitators will gather the list of questions/thematic concerns in each group and record in a flip chart In addition, each group will be provided with the pre-generated lists of potential priority areas (Additional file 2) to discuss on, augment their priorities, and identify additional priorities
Step 3. Reducing the questions and processing uncertainties	Each group will present their list of thematic concerns in a plenary session. Facilitators will create a list of unique themes by merging duplicates and overlapping questions (issues) on a flip chart. The identified themes will be grouped into key themes with list of specific concerns/issues.
Step 4. Interim Prioritization	The consolidated lists of priorities will be distributed to the homogenous groups to identify their top 10 research priorities in the order of perceived importance that they think need be the focus of research involving service users within Sodo district using pre-set criteria (e.g., relevance local primary health care and community, public health significance, magnitude of the problem, severity, feasibility/amenability to change with local context). Accordingly, each participant will select his/her top ten priorities and ranks them by giving each priority a score between 1(lowest) and 10(highest). The top list of each participant within the homogeneous groups will be combined by consensus and presented in a plenary session for listing the 10 priorities considered most important by all stakeholders group and reach consensus.
Step 5. Final Priority setting	The participants will be organized into nominal groups, and generate their top five priorities and rank them in orders of importance. This will follow the following five steps. a. The participants will be divided into four groups with balance of service user, caregivers, health professionals and community stakeholders and each participant within each group will be asked to silently generate top five priorities from the top 10 lists generated. b. A round robin approach of recording of priorities will be used to collate priorities, that is, each participant in turn will be asked to read one priority off the list within each group. This priority will be written on flip chart by a facilitator of each group. c. Once all the priorities are written on the flip chart an open discussion will be conducted to allow all participants within each group to discuss, clarify, dispute and discarded or add or modify a priority within their groups. d. The final lists of priorities from each group will be presented in a plenary session, bring similar priorities together on a flip chart, and will be discussed with the whole group in order to ensure that all participants understand and approve of the congregated priorities. e. Finally, each participants will be provided with the combined consensus priorities and asked individually and anonymously, to rank all the five most important priorities in the order of importance by giving five to the highest valued priority, the next most important, a value of four and so on progressively down to the least important which will be assigned a value of 1. A mean priority score for each priority across all groups will be calculated by summarising ranking scores and dividing this by the maximum possible ranking score of that priority. The maximum possible ranking score for a given priority will be calculated by multiplying the number of participants who considered the priority by 5(the maximum rank) Similar NGT will be conducted with RPG at a health facility level to identify and establish two top priorities for action trial.

Although JLA enables the identification of potential priority problems of interest to stakeholders; there is a need to move beyond a focus on uncertainties to the generation of shared priorities, ranking and achieve consensus on the priorities. For this, group decision making processes such as NGT are helpful, because of a well-established, multistep facilitated group interactive process through increased engagement of relevant stakeholders(including those otherwise excluded groups)on concerns that are important and matter to them [136, 137]. The processes of silent generation of responses, round-robin listening and independent voting ensures the participation of all individuals. The structure of voting and discussion allows the person to express a view, influence decisions, avoid conformity or social pressure, and individual judgments can be aggregated into group conclusions whereby anonymous individual rank-orderings are aggregated across members to determine the relative importance of all responses [136, 137]. For the proposed study, we integrated NGT with stage 5 in JLA which seems ideally suited for PAR and consistent with the critical social theory, NGT will give all participants a voice, and produce priorities and practical change [137, 138]. Following the NGT, the participants will generate, revise, vote on and rank priority problems of importance to their local context (See Table 1 for details). Participants will rate the importance of the thematic concerns on a 5-point Likert scale (very high priority to very low priority).The priority list that is created will be grouped into broad thematic areas of domains of mental health systems (e.g., research, quality improvement, advocacy) by consensus and using an inductive approach.

## STAGE 2. Plan of action development

In stage 2, the key themes generated and prioritized from Stage 1 will be presented back to the RPG and reviewed at a healthcare facility in Sodo district. The RPG will discuss the prioritized areas of concern and choose one concrete theme/problem to be addressed in their specific health facility/setting, identify lists of strategies to solve the problem and develop a plan of action for that specific health facility/local setting. During this stage, the cycles of PAR (including planning, acting, observing and reflecting) will be undertaken (See Table 2).

**Table 2:** Summary of stages, activities, and session plans for the proposed study

Stages	Cyclical activities	Descriptions	Sessions
Stage 1: Formation of stakeholders Groups, and Consultative Workshop	Planning	Identify and establish cross-stakeholder groups that services as a reference group, and working group together with Sodo district health office Getting stakeholder groups and agree on time and place for regular sessions Develop summary of findings from foundational studies Identify and prioritize top thematic concerns	1
	Action	Present and discuss on foundational studies in a consultative workshop with stakeholder groups Systematically identify thematic concerns through small homogenous groups and heterogeneous group discussions, prioritize thematic concerns using Nominal Group Techniques	2
	Observing	Collect key thematic concerns and priorities generated in small group and plenary sessions, through audio-recording, capture minutes, field notes A research assistant will record field notes on group dynamics and interactions and on the context surrounding the discussion.	1–2
	Reflection	Discuss on and reach consensus on priority areas Reflect within homogeneous groups, heterogeneous; compare the reports of each group The stakeholder groups make sense of what has happed through thinking about how it fits with their experiences and local contexts using criteria	2
Stage2: Planning of action	Planning	Reach common understanding between RPG and the researchers and assistants what the research involves and ensure consent to participate RPG agree on time, place, number of sessions per week and duration of the sessions at primary health facility level Review the thematic priorities identified in Stage 1, discuss, select and prioritize two thematic concerns for action as trial of proof of concepts Generate set of solutions and design intervention strategies	3
	Action	Work with RPG and develop viable and realistic change strategies taking into account their local realities; set evaluation strategies for actions	4
	Observing	Observe and document the process through notes, and audio-recordings Evaluate participation and representation	3–4
	Reflection	Continuous reflection throughout the action planning phases on data from observation, field notes and reflect on the action options Examine whether the proposed improvement methods is feasible in terms, time, additional resources availability, and local experiences	3–4
Stage3: Implementation and evaluation	Planning	Review of the plan action with RPG and reach agreement about the way strategies would be put into operation and how to document observations Designing implementation strategies and action Discuss about and set implementation indicators Discuss and research consensus how the RPG will continue with the PAR processes on own	5
	Action	Implementation meeting with RPG Reach agreement about the way the program would be put into operation and how to document observations Select few interventions and commence as trial of proof of concept Discuss and research consensus how the RPG will continue with the PAR processes on their own	6
	Observing	Document the trial process through taking detailed field notes, observation and discussion with RPG Preliminary analysis and findings of the process will be collected Conducted in-depth interviews with RPG to ascertain their perceptions and experiences of the process of PAR	6
	Reflection	Conduct evaluation meeting with RPG and collect feedback about the process of the PAR process, and reflect on the process of implementation Identify options for further PAR and action with or without academic researchers	7

## Stage 3: implementation and evaluation

The focus of this stage is actual field implementation of the proposed strategies and action priorities in Stage2, and evaluation of the process. In partnership with the RPG, assessment of the local context of the health facility will be conducted by the principal investigator, including identification of potential opportunities and barriers to implementing the agreed actions. The entire implementation process will be underpinned by the cyclical PAR activities of planning, acting, observing and reflection (See Table 2). However, as this study is also of interest for academic purposes (principal investigator), the authors anticipate time pressure may hinder the full involvement of the principal investigator in the final cycles. Hence, some key strategies and actions that can be implemented within the time frame of the study and will be identified during the initial discussions with RPG, implemented and evaluated(See Table 2) as a proof of concept. As the action stages evolve, the RPG will be empowered to become autonomous to take actions and effectively implement their action plan in their areas of priority, and the role of the principal investigator will become advisory and consultative [141].

## Data collection and analysis

Multiple sources and methods will be used to collect data. All participants (RAG and RPG) will fill out socio-demographic questionnaires at the beginning of the first session. The following types of data will be collected: meeting minutes, written documentation of prioritization and consensus processes, reflective field notes(reflective journal) of obstacles and successes of the research process, participant observation during all group discussions, and anonymous feedback from the participants about the process, and audio recordings of all sessions. After the last session of PAR, in-depth interviews will be conducted with the RPG members to explore their experiences of involvement in the PAR processes.

Thematic analysis of the data will be conducted [142]. The data analysis method will be based on Interpretative Phenomenological approach, which places the participants’ experiences at the core [143]. We expect that it will be challenging to involve service user in the data analysis, in the true sense of the word, hence the principal investigator will lead the data analysis, and the results of the analysis will be fed back to the participants for member checking.

### Rigour

Several measures will be employed to increase the rigour, authenticity, and trustworthiness of the proposed data collection and analysis. Bias in data collection and coding by the principal investigator and research assistant will be reduced through regular discussions(to maintain reflexivity)with the RPG [144, 145]. In addition, the principal investigator will acknowledge and record sources of potential personal bias that may influence the process of data collection and analysis as a result of existing networks and connections. The process will provide an audit trail of the reflective process; compliance with the criteria of confirmability data will be ensured by audio recording of discussion groups [144, 145]. Increased credibility will be achieved through prolonged engagement of the principal investigator within the setting, triangulation of multiple data sources and methods(e.g., written minutes, observations, field notes, and in-depth interviews), and regular member checking of raw data, and reports, which will support sustained dialogue with participants, and development of authentic, trusting rapport between the researcher and participants [144, 145]. Transferability of the study will be increased through sufficient and rich contextual description of the study setting, thick contextual data and activities details, and proper data documentation to allow others to analyze the situation and research outcomes based on setting and context [145].

### Strengths and limitations

The use of PAR to pilot the model for service user involvement within the health systems is a new experience, and to our knowledge, there have not been any studies that have piloted and evaluated ToC in conjunction with PAR for service users and caregiver involvement in mental health systems strengthening in Ethiopia or other LMICs. The PAR approach will enable us to improve the model to fit the needs of service users and improve its relevance; the co-design of the model also ensures its local applicability and sufficient adaptability to be transferable to other health facilities in LMICs. The use of PAR that embedded within critical social theory provides a strong theoretical foundation, which bring stakeholders together to define for themselves their needs and experiences, identify any areas of concern, develop a plan of action, and support the implementation of solutions. The findings of the study are likely to result in an increased understanding of complex phenomena of service user involvement; can contribute in refining the ToC model for better transferability, and may provide future researchers with useful insights and foresights in the development and implementation of more stakeholder inclusive initiatives for service user involvement in mental health systems strengthening in Ethiopia and other similar LMICs.

There are several limitations to the proposed study. The proposed pilot study is a small-scale exploratory study and there is no comparison group. A comparative study of larger scale of the proposed study is justified for interested researchers for more rigorous evaluation to provide further support to the impact of the implementation of service user/caregiver involvement experiences. The selection of the participants and pilot site is purposive and so this limits the transferability of the study findings. However, the aim of our pilot study was not to be representative of the whole landscape of primary care clinics and service-users, but to co-design a model with service-user, caregivers, primary health care professionals and health facility managers. The low literacy levels of service users and caregivers may be a barrier to involvement in all stages of the PAR process.

## Supplementary information

**Additional file 1.** Inclusion criteria for cross-stakeholder participants in the proposed study.

**Additional file 2.** Potential priority concerns to strengthen mental health systems involving service users.

## Data Availability

Not applicable.

## Abbreviations

CAB: Community Advisory Board
CST: Critical Social Theory
Emerald: Emerging mental health systems in low- and middle-income countries
HEWs: Health extension workers
JLA: James Lind Alliance
LMICs: low- and middle-income countries
NGT: Nominal Group Technique
PAR: Participatory Action Research
PRIME: PRogramme for Improving Mental health carE
RAG: Research Advisory Groups
RPG: Research Participant Groups
ToC: Theory of Change
