# Supplementary material for: Participatory action research to pilot a model of mental health service user involvement in an Ethiopian rural primary healthcare setting: study protocol
Source: Res Involv Engagem. 2020 Jan 8;6:2. doi: 10.1186/s40900-019-0175-x (PMC6951014; doi:10.1186/s40900-019-0175-x)
Supplement: Supplementary file 1 — Additional file 1. Inclusion criteria for cross-stakeholder participants in the proposed study. [file 40900_2019_175_MOESM1_ESM.docx]

Additional file 1. Inclusion criteria for cross-stakeholder participants in the proposed study

| Inclusion criteria | Stakeholder groups | | | |
| --- | --- | --- | --- | --- |
|  | Service users | Caregivers | Health professionals | CAB members |
| Motivated, willing to participate in the PAR process | X | X | X | X |
| Participated in ToC development or capacity building training or community consultative meetings | X | X | X | X |
| Health professional who take care of service users directly, those who see service users most often to provide assistance, supporting care and physical care |  |  | X |  |
| Need be from relevant statutory or non- statutory leaders (health, justice, policy, microfinance, cooperatives, social and labor affairs, women and children, faith and religious organizations, non-governmental organizations) or activists | X | X | X | X |
| Service users with mental health conditions including psychosis or epilepsy or alcohol use disorder or depression who receive mental health service within the district | X |  |  |  |
| Caregivers who are the immediate care to the service users and responsible; need be closely related with service users |  | X |  |  |
| Age 18 years and above | X | X | X | X |
| Need be valuable, committed an agreement to participate at the sessions that require their presence | X | X | X | X |
| Has a fairly stable health situation | X |  |  |  |
| To be included in RPG working or receiving services currently at or with walking distance of a primary healthcare facility for piloting the ToC model | X | X | X |  |
| Track records and potential contribution for service user and caregiver empowerment |  |  | X | X |
| Able to hold a conversation without dependence of others |  |  | X | X |
