# Supplementary material for: Participatory action research to pilot a model of mental health service user involvement in an Ethiopian rural primary healthcare setting: study protocol
Source: Res Involv Engagem. 2020 Jan 8;6:2. doi: 10.1186/s40900-019-0175-x (PMC6951014; doi:10.1186/s40900-019-0175-x)
Supplement: Supplementary file 2 — Additional file 2. Potential priority concerns to strengthen mental health systems involving service users. [file 40900_2019_175_MOESM2_ESM.docx]

Additional file 2. Potential priority concerns to strengthen mental health systems involving service users

Sources use: Qualitative study (1), ToC (2), Capacity building training (3), community stakeholder consultative meeting (4), literature review (5)

| Potential themes | List of questions or topic areas | Source of the evidence | | | | |
| --- | --- | --- | --- | --- | --- | --- |
|  |  | 1 | 2 | 3 | 4 | 5 |
| Medication | What are the ways of gaining more choices and control over the types and amount of medication use?  What are the experiences and management of medication side effects?  How availability and affordability of medication will be supported? | X | X | X | X |  |
| Developing Peer-to-peer support | How to develop, support, and promote peer-to-peer support works for mental health service user/caregivers?  How to mobilize peer workers (how to recruit, train, where to embedded and support) for mental health service delivery?  How the health care organization recruit, training and support peer-to-peer support for mental health service users/caregivers  How can health organization/facilities mobilize peer workers to support mental health service delivery?  What experiences of peer-to-peer support or support networks are there in Sodo district or somewhere else to adapt for mental health conations?  What are the most effective self help and self-management resources, approaches or techniques available for service users? | X |  | X | X | X |
| Public education and awareness campaigns | How to raise public awareness about mental health, mental illness and people with mental health conditions? | X | X | X | X |  |
| Service user involvement | How can service users and caregivers perspectives be incorporated into service planning, service delivery, quality improvement, professionals training?  How can service user/caregivers be more involved in making-decisions about their mental healthcare in a health facility?  How to develop service user/caregiver in advocacy for promoting their rights?  How service user/caregiver be involved in making health service person-centered?  How can service user/caregivers be more involved in public awareness raising?  How can service user/caregiver led-organizations promoted? | X | X | X | X | X |
| Stigma and discrimination | How service user/caregivers and their organization can be supported to overcome stigma and discrimination within the healthcare organizations and community?  How can service user/caregivers can be part of promoting social inclusion of service user?  How health organization/professionals can work with other community stakeholders (CAB) to ensure service users are not excluded  How to reduce the use of unhelpful languages and labels that stigmatise service users? | X | X | X | X | X |
| Improving quality of health and health care | How to identify and promote respectful and helpful relationship between service user and caregivers?  How can service user, caregiver, and health professionals, best work together to promote respectful, compassionate person-centered healthcare improve the quality of support and choice of care within primary healthcare for service user/caregivers?  How can health care organizations, community stakeholder organizations work together in a more effective manner in order to improve the socio-economic conditions of service users? | X | X | X | X | X |
| Early diagnosis and treatment care pathways and transitions between services | How service user/caregivers can be involved to encourage people with mental health conditions to receive timely diagnosis and treatment?  What are the best ways to support service users and their caregivers where there are alcohol use problem?  How to effectively and acceptably work with healthcare organizations/professionals, and traditional and religious healers to shorten care pathways to improve service users access to appropriate healthcare at the right time  How do we educate people to recognize early warning signs of mental illness prior to crisis and seek help before an illness become more severe?  What are the mental health service pathways (traditional, religious and modern) experiences of service users and their caregivers to inform decisions about the most appropriate care pathways? | X | X | X | X | X |
| Interface between mental health conditions and physical health( Commorbidity) | How to meet the socioeconomic and psychosocial needs of service users within primary healthcare?  How are service users and caregivers experiencing mental illness and alcohol use?  What role service users and caregivers can play in reducing alcohol use in people with mental health conditions? | X | X | X | X | X |
| The importance of socio-environmental factors | What contribution do support networks and peer support have on mental health?  What are the effects of financial insecurity on mental h ill health?  What contribution do support networks and peer support have on mental health?([1](file:///C:\Users\user\Desktop\Draft%20protocol\Additional%20file%204-1.docx#_ENREF_1)) | X | X | X | X | X |
| Stakeholder collaboration | How can we improve local community collaboration to mobilize local social and cultural assets, networks/community structures, and resources to meet the socio-economic and psychosocial needs of service users and caregivers within primary health care?  How can service user, caregivers, health professionals and other key community stakeholder work together in order to improve service users and caregivers involvement in mental health systems improvement? | X | X | X | X | X |
| Information needs /service coordination | What are the best ways to facilitate communication across services and between healthcare professionals? |  |  |  |  |  |
| Service user empowerment | Organizing ,resource mobilization  Establishing user associations and caregivers association  How organize, moblise resources, support and empower caregiver and service users  Service user association initiatives and the role they play in service user and caregiver centered services  What are the best ways of supporting service users to manage and live with mental illness? |  |  |  |  |  |
| Experiences of mental health condition | Impact on life, how to live with mental ill health and related support issues |  |  |  |  |  |
| Improving Treatment and social conditions | What are the best care packages for service users, caregivers which combine healthcare and social care and take individual prognosis into consideration?  How to improve social and economic circumstances(poverty, housing, education and employment)  How to improve access to effective non-pharmacological treatments |  |  |  |  |  |
| Raising awareness (policy makers, general public, caregivers) | Promoting understanding and acceptance: promote community awareness and education about metal illness to minimize stigma and discrimination  Prevention and mental health promotion  Social inclusion and the role of mental health and social care |  |  |  |  |  |
| Empowering self--management | Gaining knowledge and skill to support self-management  Motivation for treatment adherence: strategies that support and encourage positive behaviors in service user regarding treatment adherence  Service users contributions to their own safety  Effectiveness of self management techniques  What are the most effective ways of supporting service users to manage their condition on their own? |  |  |  |  |  |

References

1. Robotham D, Wykes T, Rose D, Doughty L, Strange S, Neale J, et al. Service user and carer priorities in a Biomedical Research Centre for mental health. Taylor & Francis; 2016.

2. Banfield M, Randall R, O'Brien M, Hope S, Gulliver A, Forbes O, et al. Lived experience researchers partnering with consumers and carers to improve mental health research: Reflections from an Australian initiative. International journal of mental health nursing. 2018.

3. Banfield MA, Morse AR, Gulliver A, Griffiths KM. Mental health research priorities in Australia: a consumer and carer agenda. Health research policy and systems. 2018;16(1):119.

4. Whiston L, Barry J, O’Keane V, Darker C. Patient, family and clinician preferences for the intensity and implementation of patient and family participation in healthcare design and delivery in psychiatry. The European Journal of Psychiatry. 2018.

5. Rose D, Fleischman P, Wykes T. What are mental health service users' priorities for research in the UK? Journal of Mental Health. 2008;17(5):520-30.

6. Thornicroft G, Rose D, Huxley P, Dale G, Wykes T. What are the research priorities of mental health service users? Journal of mental health. 2002;11(1):1-3.

7. James P, Aitken P, Bums T. Research priorities for primary care mental health: a Delphi. Primary Care Psychiatry. 2002;8(1):27-30.

8. Naylor C, Samele C, Wallcraft J. Research Priorities for ‘Patient-Centred’Mental Health Services: Findings From a National Consultation. Mental Health Review Journal. 2008;13(4):33-43.

9. Emrich-Mills L, Hammond LL, Rivett E, Rhodes T, Richmond P, West J. Identifying research priorities for older people’s mental health services. Mental Health and Social Inclusion. 2019.

10. Hollis C, Sampson S, Simons L, Davies EB, Churchill R, Betton V, et al. Identifying research priorities for digital technology in mental health care: results of the James Lind Alliance Priority Setting Partnership. The Lancet Psychiatry. 2018;5(10):845-54.
